# Amyloid-β and caspase-1 are indicators of sepsis and organ injury

**DOI:** 10.1183/23120541.00572-2023

**Published:** 2024-02-26

**Authors:** Amanda N. Tuckey, Arcole Brandon, Yasaman Eslaamizaad, Waqar Siddiqui, Talha Nawaz, Christopher Clarke, Erica Sutherland, Veronica Williams, Domenico Spadafora, Robert A. Barrington, Diego F. Alvarez, Madhuri S. Mulekar, Jon D. Simmons, Brian W. Fouty, Jonathon P. Audia

**Affiliations:** 1 Department of Microbiology and Immunology, University of South Alabama College of Medicine; 2 Center for Lung Biology, University of South Alabama College of Medicine; 3 Department of Internal Medicine, University of South Alabama College of Medicine; 4 Division of Pulmonary and Critical Care Medicine, University of South Alabama College of Medicine; 5 Department of Laboratory Medicine, University of South Alabama University Hospital; 6 Flow Cytometry Shared Resources Laboratory, University of South Alabama College of Medicine; 7 Department of Pharmacology College of Medicine, University of South Alabama College of Medicine; 8 Department of Mathematics and Statistics, University of South Alabama College of Arts and Sciences; 9 Department of Surgery, University of South Alabama College of Medicine

## Abstract

**Background:**

Sepsis is a life-threatening condition that results from a dysregulated host response to infection, leading to organ dysfunction. Despite the prevalence and associated socioeconomic costs, treatment of sepsis remains limited to antibiotics and supportive care, and a majority of intensive care unit (ICU) survivors develop long-term cognitive complications post-discharge. The present study identifies a novel regulatory relationship between amyloid-β (Aβ) and the inflammasome-caspase-1 axis as key innate immune mediators that define sepsis outcomes.

**Methods:**

Medical ICU patients and healthy individuals were consented for blood and clinical data collection. Plasma cytokine, caspase-1 and Aβ levels were measured. Data were compared against indices of multiorgan injury and other clinical parameters. Additionally, recombinant proteins were tested *in vitro* to examine the effect of caspase-1 on a functional hallmark of Aβ, namely aggregation.

**Results:**

Plasma caspase-1 levels displayed the best predictive value in discriminating ICU patients with sepsis from non-infected ICU patients (area under the receiver operating characteristic curve=0.7080). Plasma caspase-1 and the Aβ isoform Aβx-40 showed a significant positive correlation and Aβx-40 associated with organ injury. Additionally, Aβ plasma levels continued to rise from time of ICU admission to 7 days post-admission. *In silico*, Aβ harbours a predicted caspase-1 cleavage site, and *in vitro* studies demonstrated that caspase-1 cleaved Aβ to inhibit its auto-aggregation, suggesting a novel regulatory relationship.

**Conclusions:**

Aβx-40 and caspase-1 are potentially useful early indicators of sepsis and its attendant organ injury. Additionally, Aβx-40 has emerged as a potential culprit in the ensuing development of post-ICU syndrome.

## Shareable abstract

**Aβx-40 and caspase-1 are potentially useful early indicators of sepsis and attendant organ injury, and caspase-1 cleavage of Aβ alters its functional properties, thus suggesting a novel regulatory relationship** https://bit.ly/489n0Da

## Introduction

The Centers for Disease Control and Prevention estimate the occurrence of 1.7 million sepsis cases per year in the United States. Severe cases progress to septic shock, characterised by profound inflammatory, circulatory and metabolic dysfunction frequently leading to organ failure and death [1–3]. Despite advances in supportive care and early antibiotic therapy, the ∼270 000 sepsis-associated deaths per year account for one-third of all in-hospital mortality [2]. High morbidity and mortality is due in part to the paucity of diagnostics to identify sepsis patients early and intervene to prevent the attendant systemic dysfunction. Compounding the problems related to acute sepsis-associated morbidity, approximately half of survivors develop post-sepsis syndrome with inflammation-driven complications.

Amyloid-β (Aβ) has recently emerged as a pleiotropic innate immune effector [4–6]. The APD3 Antimicrobial Peptide Database classifies Aβ as an antibacterial, antiviral and antifungal peptide that entraps and neutralises invading pathogens [7]. Additionally, Aβ is also a known culprit of inflammation *via* pattern recognition receptor activation, including toll-like receptor 2 [8] and the NLRP3-caspase-1 inflammasome [9–11]. Thus, factors that dysregulate the innate immune function of Aβ during infection may lead to downstream activation of potentially deleterious inflammation. While potential roles for Aβ in sepsis have been studied in animal models, there is a paucity of studies in humans [12, 13].

With sepsis being characterised as a dysregulated host response to infection, we hypothesised that plasma Aβ levels would correlate with sepsis severity. To test this, we conducted a prospective study comparing plasma analyte levels of Aβ and other cytokines in healthy individuals to patients in the intensive care unit (ICU) with or without sepsis.

## Methods

### Study design and population

This prospective study was conducted using plasma samples collected between 2015 and 2021 from healthy control individuals and from patients admitted to the University of South Alabama University Hospital (University Institutional Review Board approvals 687050-15 and IRB # 20-443). The target population was 19–64-year-old individuals admitted to the medical ICU. Upon admission, patients were enrolled, informed consent obtained (patient or designated decision maker), and blood obtained from an in-dwelling catheter or by venipuncture. For healthy controls, blood was collected by venipuncture. Of the 289 participants enrolled, 176 (61%) were healthy controls and 113 (39%) were ICU patients. A total of 77 out of 113 ICU patients (68%) were classified as septic. Of note, our study was initiated prior to the Sepsis-3 criteria [14], and therefore, the attending physician utilised the Sepsis-2 criteria to identify individuals for enrolment [15, 16]. A summary of patient demographics and clinical data are presented in table 1, building on a subset of these data that were previously reported [17]. Of the ICU patients, 14 out of 36 (39%) of the non-septic and 38 out of 77 (49%) of the septic patients also had blood collected at 7 days post-admission (7dp) to the ICU (in two BD Vacutainer™, ThermoFisher #02685125). Blood was processed within 1 h of acquisition as per the manufacturer's directions. Plasma was aliquoted (1 mL per cryovial) and stored at −80°C until assayed. Within the septic cohort, patients were further characterised based on the origination of the infection, as determined by the attending physician (supplementary figure S2).

**TABLE 1:** Patient demographics of University of South Alabama ICU patients

**Characteristic**	**Healthy controls**	**ICU-non-sepsis^#^**	**ICU-sepsis**
**Patients, n**	176	36	77
**Age years, mean (range)**	38 (22–76)	55.7 (21–82)	58.9 (20–91)
**Male sex, n (%)**	55 (31.3)	16 (44.4)	44 (57.1)
**Female sex, n (%)**	121 (68.7)	20 (55.6)	35 (45.5)
**Race, n (%)**			
** **White	137 (77.8)	19 (52.8)	35 (45.5)
** **Black	33 (18.8)	17 (47.2)	42 (54.5)
**Intubated, n (%)**	N/A	7 (19.4)	32^¶^ (41.6)
**Vasopressor, n (%)**	N/A	3^+^ (8.6)	21 (27.3)
**SOFA score, mean**	N/A	2.74	5.47
**Days in hospital, mean**	N/A	9.03	16.55
**Days in ICU, mean**	N/A	3.15	6.13
**GCS, mean**	N/A	12.91	10.42
**Mortality in ICU, n (%)**	N/A	1 (2.78)	18 (23.4)

SOFA: Sequential (Sepsis-Related) Organ Failure Assessment; GCS: Glasgow Coma Scale; ICU: intensive care unit; N/A: not applicable. ^#^: stroke and seizure/encephalophagy accounted for 50% and 20% of ICU-non-sepsis patients, respectively; ^¶^: intubation status of two sepsis patients are unknown; ^+^: vasopressor status of one ICU non-sepsis patient is unknown.

### Plasma cytokine, caspase-1 and Aβ measurements

Plasma samples were thawed on ice and centrifuged (18 000 × *g*, 4°C, 5 min) to remove particulates. Supernatants were diluted 1:4 for assay using the Human Inflammation Panel 1 (13-plex) as per the manufacturer's directions (BioLegend LEGENDplex, 740808). Data were acquired *via* flow cytometry (BD FACS Canto II) and processed using FlowJo software (TreeStar). Caspase-1 was quantified using undiluted supernatant and a Caspase-1 ELISA (Quantikine, DCA100). The ELISA plates were analysed on a microplate reader (BioTek EPOCH 2) at 450–560 nm. Aβ isoforms 38, 40 and 42 were quantified using the Mesoscale Discovery (MSD) Multi-Spot Aβ (4G8) Peptide Assay (K15199E). Post-centrifugation, plasma samples were equilibrated to ambient temperature, diluted 1:4 in diluent 35 and assayed as per the manufacturer's directions. MSD plates were analysed on the MESO QuickPlex SQ and the data processed using the MSD Discovery Workbench software. All assay values outside the limit of detection were termed ND and excluded from analysis. Plasma analyte analyses were performed for all ICU patients and for 55 randomly selected healthy controls.

### Thioflavin T assay

Monomeric Aβ peptides spontaneously oligomerise to form fibrils, which is measured kinetically *via* intercalation of Thioflavin T (ThT) [18]. ThT powder (Acros Organics 211760050) was reconstituted to 2.5 mM in absolute ethanol and stored at −20°C protected from light. Recombinant human monomeric Aβx-40 and Aβx-42 (Millipore AG962 (Aβ1-40) and AG968 (Aβ1-42)) were reconstituted to 222.1 µM in 1% NH_4_OH and stored at −80°C. Recombinant human caspase-1 (BioVision 1081-100) was reconstituted in ultra-pure water to 1 unit·µL^−1^ and stored at −80°C. Reactions were prepared by combining ThT (10 µM final) and monomeric Aβ (5, 10 or 20 µM final) in phosphate-buffered saline (PBS, Ca^++^/Mg^++^-free, pH 7.4). Experimental reactions contained ThT, monomeric Aβ, and caspase-1 (5 or 10 units·mL^−1^). All reactions were run in duplicate with at least three biological replicates. Fluorescence was measured kinetically at 37°C using a BioTek Synergy 2 plate reader with filter set Ex=420/27, Em=485/20. Data were normalised to a negative control reaction containing ThT in PBS alone (background was subtracted for each time point).

### Statistics

Statistical analyses were performed using software JMP Pro v 16.2.0 (a product of SAS, Inc., Cary, NC, USA) or GraphPad Prism v9. A majority of analytes measured displayed a high variance with a skew towards high-value outliers (supplementary tables S1 and S2), therefore, medians were used for comparisons between groups. A p-value ≤0.05 was considered significant. Correlations between numerical variables were determined using Pearson's correlation coefficient. Variations in measurements within three groups were compared using Levene's test. Mean outcomes in three groups were compared using Welch's ANOVA taking into account differences in variations. The median test was used to compare median outcomes for two or three groups. Dunnett's test was used for *post hoc* analysis with healthy individuals as a control, *i.e.*, the remaining two groups (ICU-non-sepsis and ICU-sepsis) were compared with healthy controls. Tukey's HSD was used for *post hoc* analysis comparing all three groups. The non-parametric Steel test was used for comparison with control, or the Steel–Dwass test was used for *post hoc* comparison of all three groups. One-sample z-test for proportion was used to determine if a significantly higher percentage of measurements in non-control groups exceeded median level of the control. Fisher's exact test was used to compare outcome proportions of two groups. To investigate the predictive capacity of the analytes, we utilised the receiver operating characteristic (ROC). In brief, non-sepsis patients were used as the “control” and compared against the sepsis “patient”. With this method, we demonstrate the diagnostic ability of the analyte to correctly classify the ICU patient.

## Results

This prospective study examined three cohorts: healthy individuals, ICU patients with sepsis (ICU-sepsis) and ICU patients without sepsis (ICU-non-sepsis). The underlying rationale for enrolling an ICU patient cohort without suspicion of sepsis was to augment the potential for identifying plasma analytes specifically affected by infection. The ICU patients, with or without sepsis, were similar in mean age, sex and race distribution (table 1). In the ICU-non-sepsis cohort, stroke (50%) and seizure/encephalopathy (20%) were the predominant diagnoses.

### Caspase-1 is significantly elevated in plasma of sepsis patients

**FIGURE 1:**
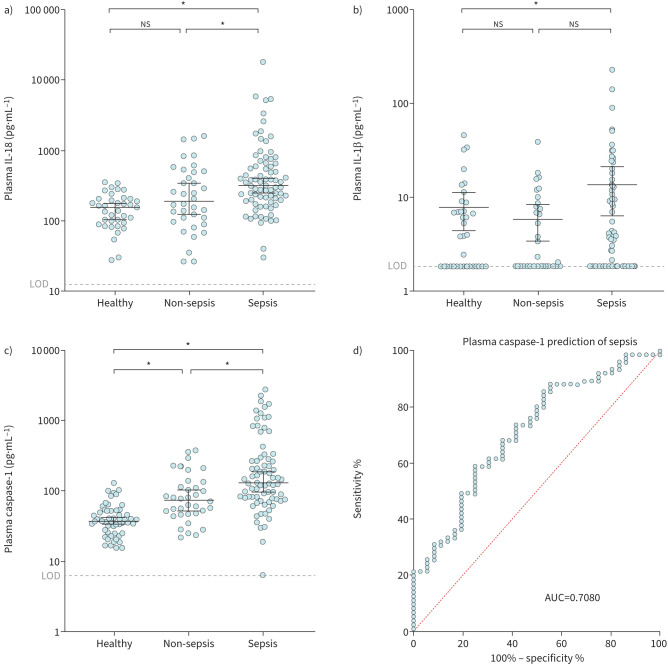
Caspase-1 plasma levels were significantly elevated in all ICU patients and were an accurate predictor of sepsis. a) Interleukin (IL)-18 and b) IL-1β were elevated in sepsis patients, an observation consistent with previously published work. c) Plasma caspase-1 levels were elevated in both intensive care unit cohorts, and were highest in the sepsis cohort. d) Receiver operating curve (ROC) analysis using non-sepsis as the control and sepsis as the patient group (area under the curve (AUC) 0.7080). ns: nonsignificant. *: p<0.05 was considered significant when comparing medians using a Steel test.

Similar to previous studies [19–21], our ICU-sepsis group displayed significantly increased interleukin (IL)-18, IL-1β (figure 1a and b), IL-6, CXCL-8, IL-10 and MCP-1 compared to either or both of the ICU-non-sepsis patient and healthy cohorts (supplementary figure S1). During infection and sepsis, the NLRP3-inflammasome initiates pro-caspase-1 activation *via* auto-proteolysis [22–24]. Active caspase-1 is a pro-inflammatory cysteine active site aspartate-specific protease that cleaves pro-IL-1β and pro-IL-18 into their active forms (reviewed in Franchi *et al.* [25]). Indeed, caspase-1 was also significantly elevated in both ICU groups compared to healthy controls with overall levels being highest in the ICU-sepsis patients (figure 1c). The elevation of capsase-1 in the sepsis group was independent of whether the infection was of pulmonary or extrapulmonary origin (supplementary figure S2a). Caspase-1 was a highly accurate indicator of sepsis (area under the ROC=0.7080, figure 1d).

### Aβx-40 is significantly elevated in sepsis patient plasma

**FIGURE 2:**
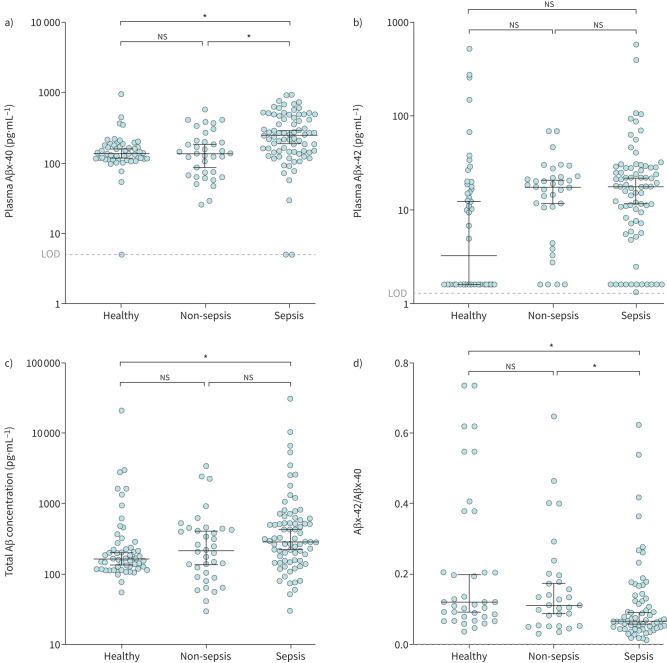
Aβ isoform Aβx-40 and Aβx-42/Aβx-40 plasma levels were significantly elevated in intensive care unit (ICU) patients with sepsis. a) Aβx-40 and d) the Aβx-42/Aβx-40 ratio are significantly elevated in the sepsis cohort compared to both the healthy and ICU-non-sepsis cohorts. c) Total Aβ is significantly elevated in the sepsis cohort compared to the healthy cohort. b) Aβx-42 was not significantly different between any cohorts. ns: nonsignificant. *: p<0.05 was considered significant when comparing medians using the Steel test.

Aβ has recently emerged as an innate immune effector that is produced in response to infection [4–6]. Sequential cleavage of the amyloid precursor protein by the β- and γ-secretase complexes generates three predominant Aβ isoforms (Aβx-38, Aβx-40 and Aβx-42) [26]. Aβ isoforms have been well characterised in Alzheimer's disease (AD) patients, in which AD patients show a reduction of plasma Aβx-40 [27, 28] to equivalent Aβx-42 levels [27], causing a decreased Aβx-42/Aβx-40 ratio. Of the three isoforms, only Aβx-40 was significantly different between any of the three cohorts (figure 2 and supplementary figure S1) and was specifically elevated in the ICU-sepsis cohort compared to the healthy and ICU-non-sepsis cohorts (figure 2a). In addition, median levels of total plasma Aβ were significantly higher in the ICU-sepsis patients compared to healthy cohorts (figure 2c). Moreover, the median Aβx-42/Aβx-40 ratio was also significantly elevated in the ICU-sepsis cohort compared to the healthy and ICU-non-sepsis cohorts (figure 2d). The elevation of Aβx-40 in the sepsis group was independent of where the infection originated (supplementary figure S2a). These results suggest that Aβx-40 is a potentially clinically useful indicator of infection and/or sepsis.

### Aβx-40 correlates with organ injury severity

**FIGURE 3:**
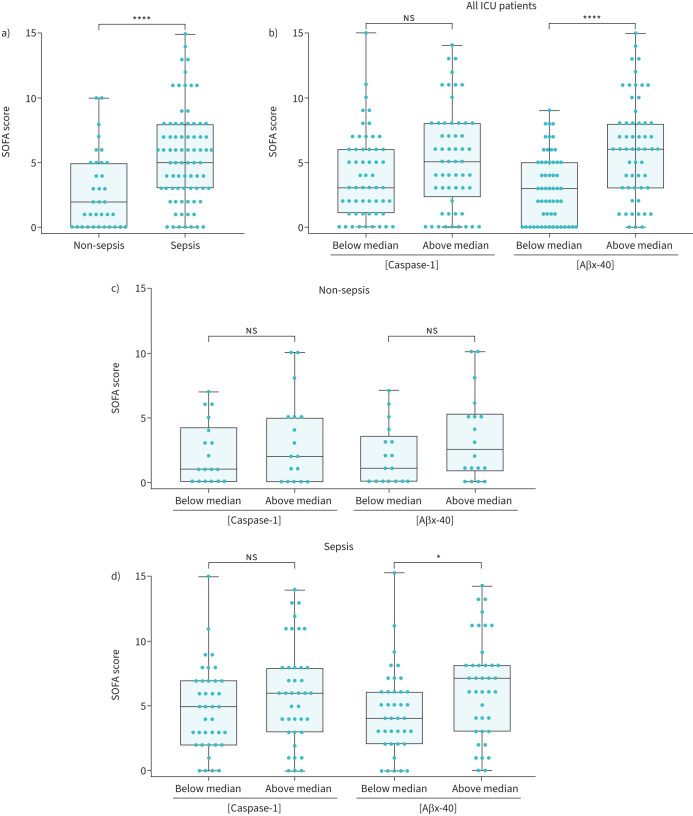
Aβ isoform Aβx-40 is an accurate predictor of organ injury. a) The intensive care unit (ICU)-sepsis cohort SOFA score is significantly higher than ICU-non-sepsis cohort. b) The ICU-non-sepsis cohort SOFA scores were not significantly different when compared by either Aβx-40 or caspase-1. c) The ICU-sepsis cohort SOFA scores were significantly increased when compared by Aβx-40 but not by caspase-1. d) Aβx-40 was a better indicator of SOFA score compared to caspase-1. SOFA: Sequential (Sepsis-Related) Organ Failure Assessment. ns: nonsignificant. *: p<0.05 or ****: p<0.001 were considered significant when comparing medians using the Mann–Whitney test.

To assess organ injury, the attending physician used the Sequential (Sepsis-Related) Organ Failure Assessment (SOFA) score guidelines [29]. The SOFA score was significantly higher in the sepsis compared to the non-sepsis cohort (figure 3a). We next determined whether caspase-1 or Aβx-40 were indicators of organ injury. Considering that ICU patient plasma levels of caspase-1 or Aβx-40 were bimodal, with distinguishable groups of high and low responders, we assessed SOFA scores based on whether plasma analyte values were below or above the median value. When all ICU patient data were concatenated and grouped based on their median level of caspase-1 or Aβx-40, only those with higher plasma Aβx-40 levels had significantly higher SOFA scores (figure 3b). Next, we separated the two ICU cohorts. Non-sepsis patients showed no significant difference in SOFA scores regardless of whether they were segregated based on median caspase-1 or Aβx-40 levels (figure 2c). However, sepsis patients with plasma Aβx-40 levels above the median had significantly higher SOFA scores (figure 3d). Conversely, there were no differences in SOFA scores when patents were analysed based on caspase-1 levels (figure 3d). Thus, plasma Aβx-40 level may be a possible indicator of organ injury severity in ICU patients. Importantly, out of all patient outcome analyses with respect to caspase-1 or Aβx-40 (supplementary figure S3), our major finding was that plasma Aβx-40 levels significantly correlated with longer ICU stays, need for vasopressors, need for haemodialysis and mortality (table 2).

**TABLE 2:** Correlations with patient outcomes

**Outcome**	**Aβx-40**	**Caspase-1**
**SOFA score**	p<0.0001 n=110	p=0.4260 (ns) n=111
**Days in ICU**	p=0.0024 n=108	p=0.5136 (ns) n=109
**Vasopressors**	p=0.0026 n=109	p=0.3164 (ns) n=110
**Mortality**	p=0.0455 n=106	p=0.2182 (ns) n=107
**Haemodialysis**	p<0.0001 n=106	p=0.1560 (ns) n=107

Correlations were drawn between all ICU patients and within each of the three groups utilising Pearson's correlation. Correlations are shown as the t-test p-value and number of pairs of observations (n). p<0.05 was considered significant. SOFA: Sequential (Sepsis-Related) Organ Failure Assessment; ICU: intensive care unit; ns: nonsignificant.

Considering that increased plasma caspase-1 was a reliable sepsis indicator and Aβx-40 was a reliable organ injury indicator, we next analysed these two markers for correlation. Indeed, Aβx-40 and caspase-1 were significantly correlated in ICU-sepsis patients but not in the healthy control or ICU-non-sepsis groups (table 3). Caspase-1 levels also correlated with its downstream effector, IL-18, in both ICU patient groups (table 3). Together, these results suggest that the combination of Aβx-40 and caspase-1 might be useful to predict sepsis and attendant organ injury.

**TABLE 3:** Correlations with caspase-1

**Caspase-1**	**Healthy controls**	**ICU-non-sepsis**	**ICU-sepsis**	**All patients**
**IL-18**	ns	r=0.41^#^ p=0.0135 n=36	r=0.39 p=0.0004 n=76	r=0.43 p<0.0001 n=149
**Aβx-40**	ns	ns	r=0.32 p=0.0051 n=74	r=0.36 p<0.0001 n=163

Correlations were drawn between all patients and within each of the three groups utilising Pearson's correlation. Correlations are shown as Pearson's r, followed by t-test p-value below, and number of pairs of observations (n). r>0.3 and p<0.05 were considered significant. ICU: intensive care unit; IL-18: interleukin-18; ns: nonsignificant. ^#^: patient plasma samples were categorised as healthy, ICU-non-sepsis or ICU-sepsis patients.

### Aβ plasma levels increase significantly from time of admission to 7dp

**FIGURE 4:**
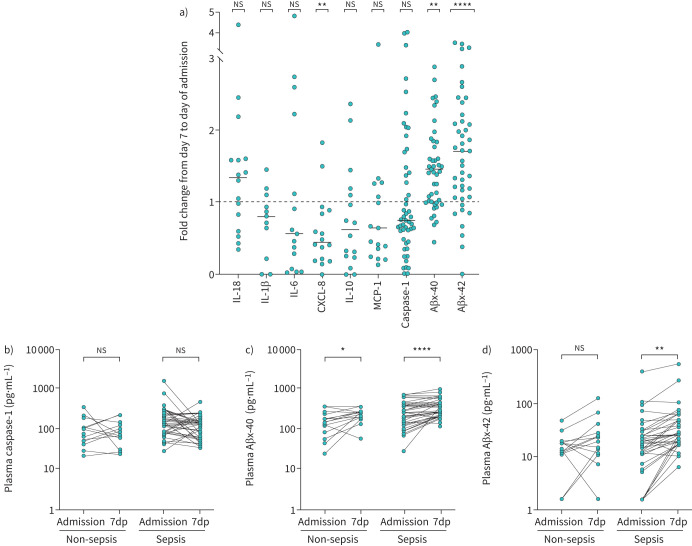
Aβ isoform Aβx-40 and Aβx-42 plasma levels increased significantly from time of admission to 7 days post-admission (7dp). a) Analyte plasma levels are represented as the fold change from 7 days post­intensive care unit (ICU) admission (7dp) compared to the levels at the time of ICU admission (Admission). CXCL-8 plasma levels were significantly reduced from time of admission to 7dp whereas interleukin (IL)-18, IL-1β, IL-6, IL-10, MCP-1 and caspase-1 plasma levels were unchanged. b) Caspase-1 plasma levels were not significantly reduced from admission to 7dp, but the levels trended towards reduction in both the non-sepsis and sepsis cohorts. a), c) and d) Aβx-40 and Aβx-42 plasma levels were significantly higher at 7dp compared to admission in the sepsis cohort, whereas Aβx-40 plasma levels were also significantly higher in the non-sepsis cohort. ns: nonsignificant. *p<0.05, **p<0.005 and ****p<0.0001 were considered significant using either a) one-sample t-test comparing the means to 1 or b–d) paired t­-test.

A 7dp admission sample was obtained for the subset of ICU patients, and we compared plasma analyte levels as fold change from admission to 7dp in both ICU groups. IL-18, IL-1β, IL-6, IL-10, MCP-1 and caspase-1 were not significantly different between the two time points, although all showed a downward trend (figure 4a). More intriguingly, plasma Aβx-40 and Aβx-42 levels significantly increased at 7dp compared to admission (figure 4a). Caspase-1 plasma levels were not significantly different in either the sepsis or non-sepsis cohorts (figure 4b), whereas Aβx-40 and Aβx-42 plasma levels significantly increased from time of admission to 7dp in the sepsis cohort (figure 4c and d). Only Aβx-40 significantly increased in the non-sepsis cohort (figure 4c).

### Caspase-1 cleaves monomeric Aβx-40 and Aβx-42 to prevent aggregation

**FIGURE 5:**
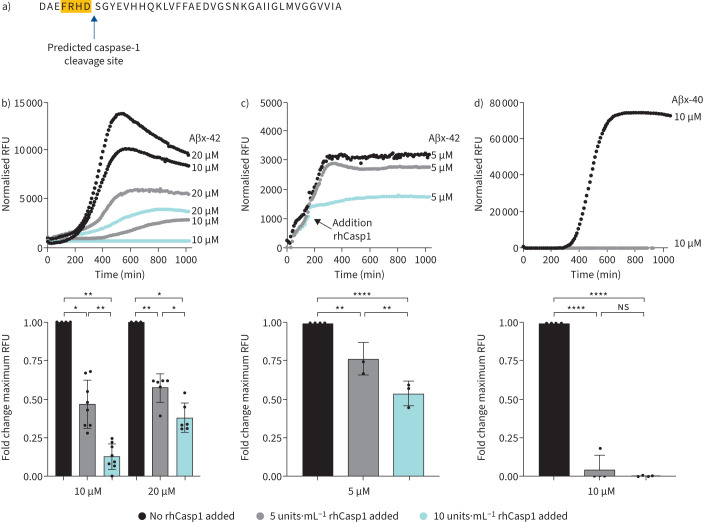
Caspase-1 inhibits Aβ isoform Aβx-40 and Aβx-42 from forming fibrils *in vitro*. a) The Aβ amino sequence harbours a predicted caspase-1 cleavage site at position 7 (FRHD) and is present in both Aβx-40 and Aβx-42. b) and c) *In vitro* biochemical reaction conditions were established to monitor Aβx-42 and d) Aβx-40 fibril formation using recombinant proteins and the fluorescent indicator dye, Thioflavin T (ThT) (expressed as relative fluorescence units (RFU)). A parallel set of reactions was tested in the presence of recombinant human caspase-1 (rhCasp1). Top panels depict kinetic data (time in minutes) and bottom panels depict the fold change in the maximum fluorescence value at the reaction peak (relative to no rhCasp1 control reactions). ns: nonsignificant. *p<0.05, **p<0.01 and ****p<0.0001 were considered significant by one-way ANOVA with Tukey's *post hoc* test.

While Aβ functionality has been best-studied in the context of amyloid plaques and neuroinflammation in AD, recent studies have highlighted its antimicrobial properties. This dual functionality of Aβ, whether pathophysiological or protective, has been tied to its capacity for spontaneous fibril formation [30]. Intriguingly, monomeric Aβ has neuronal protective properties; therefore, preventing Aβ fibril formation may protect against AD pathophysiology [30]. Moreover, Aβ is a known activator of the NLRP3-caspase-1 inflammasome that can incite neuroinflammation [10, 31–33]. Interestingly, Aβ is predicted to harbour a caspase-1 recognition site at amino acid position 7 (FRHD) (figure 5a). The presence of a putative cleavage site along with the strong clinical correlation between Aβx-40 and caspase-1 levels in sepsis patients suggested a regulatory relationship between the two. Thus, we leveraged the fact that recombinant monomeric Aβ spontaneously oligomerises to form amyloid fibrils, which can be measured kinetically *in vitro via* intercalation of the fluorescent indicator ThT. To this end, we tested the effects of recombinant human caspase-1 (rhCasp1) on Aβ fibril formation. We first validated the assay using Aβx-42 as a positive control based on its known propensity for fibril formation. Figure 5b (top panel) shows the maximum Aβx-42 fibril formation (*i.e.*, the highest fluorescence intensity (relative fluorescence units)) without any rhCasp1 present. Addition of rhCasp1 caused a dose-dependent decrease in fibril formation rate (figure 5b, top and bottom panels). More intriguingly, rhCasp1 addition after initiation of Aβx-42 fibril formation caused an almost immediate cessation of the reaction (figure 5c, top and bottom panels). We also verified that Aβx-40 fibril formation is completely prevented by addition of rhCasp1 (figure 5d). Together, these data are strongly suggestive of a regulatory relationship between caspase-1 and Aβ, specifically that caspase-1 activation opposes the formation of amyloid fibrils, which could limit its neuroinflammatory capacity.

## Discussion

Sepsis is defined as a dysregulated host response to infection caused by a diverse group of microbial pathogens entering the body through various sites. As evidenced by their highly heterogeneous inflammatory response to infection, critically ill patients constitute a complex array of “disease endotypes/subclasses” [34–36]. Our ICU patients exhibited a high degree of variance with heterogeneous outcomes in a majority of the analytes tested (supplementary Tables S1 and S2). In particular, ICU-sepsis patients were the most skewed with more high-end outliers. Highlighting this concept of variance, the data presented herein demonstrate that caspase-1 was significantly elevated and skewed towards the higher end in both ICU cohorts compared to healthy controls, and its plasma levels were a highly reliable indicator of sepsis as a primary outcome. In addition, Aβx-40 was significantly elevated and skewed towards the higher end in the plasma of ICU-sepsis patients and was a reliable organ injury indicator. Together, these data suggest that Aβ is indicative of a sepsis-specific, infection-associated ICU patient endotype, and caspase-1 is indicative of a pro-inflammatory ICU patient endotype.

Our study compares well to the limited number of other studies that have assessed sepsis outcomes using multiple biomarkers [19, 20]. In addition, caspase-1 and IL-18 were previously shown to be significantly increased in plasma and in peripheral blood cells isolated from a diverse group of critically ill patients in a multi-site ICU study [21]. Building on this previous work, our study identified caspase-1 and Aβx-40 as indicators of sepsis and end organ injury, respectively, independent of pathogen. Future studies using animal and cell culture models, in a vertically integrated reverse translation approach, will augment our understanding of the biological consequences of pathogen-induced caspase-1 and Aβx-40 elevation.

Our previous studies using cell culture and animal models have described paradoxical functions of caspase-1 activation during infection. In cultured lung endothelial cells and macrophages, pathogen-induced caspase-1 activation can be pro-inflammatory and incite damage [37, 38]. Conversely, caspase-1 can also work to protect pulmonary barrier function during infection [39]. More intriguingly, infection models have also revealed paradoxical shifts in Aβ functionality. Aβ-induced activation of the CD36 and formyl peptide scavenger receptors is mechanistically linked to neurovascular damage [40–42] and immune cell chemotaxis [43–46], respectively. In addition, lung endothelial cells produce either cytotoxic Aβ [47] or antimicrobial Aβ depending on the virulence factors that the pathogen deploys [48]. Together, these data suggest caspase-1 and Aβ can drive either salutary or deleterious effects in response to infection; however, the impact of pathogen virulence factors on the switch between these two properties remains poorly understood.

Evidence beyond our work on caspase-1 and Aβ in ICU-sepsis patients further suggest a complex regulatory relationship between the two. Aβ is a danger signal that induces expression and activation of the NLRP3 inflammasome and caspase-1 [33]. Indeed, previous studies have demonstrated that inhibition of caspase-1 ameliorates AD-related phenotypes in mouse models [49, 50]. Thus, our observation that Aβ harbours an *in silico* caspase-1 cleavage site and our *in vitro* data showing that recombinant caspase-1 prevented spontaneous Aβ fibril formation appear potentially paradoxical. The potential deleterious effects of Aβ and caspase-1-driven hyper-inflammation are undisputed. However, NLRP3-caspase-1 inflammasome activation by other damage-associated molecular patterns released in the context of AD will trigger deleterious inflammation, hence the observations that caspase-1 inhibition ameliorates AD. Thus, our biochemical data suggest that Aβ and caspase-1 form a regulatory feedback loop whereby caspase-1 cleavage of Aβ may function to temper NLRP3-caspase-1 inflammasome over-activation. This regulatory loop is not likely an “all-or-nothing” response and may indicate there are other consequences related to caspase-1 cleavage of Aβ. Thus, it will be critical to untangle other Aβ functional changes induced by caspase-1 cleavage and whether this relationship defines their switch between salutary or deleterious effects in response to infection.

The potential impact of our observations extends beyond sepsis. The deleterious effects of Aβ-mediated inflammation underlie its potential causative role in AD and AD-related dementias *via* microglial activation leading to blood–brain barrier dysfunction [51]. Thus, our discovery that Aβx-40 and caspase-1 are coordinately elevated during sepsis suggests their potential to impact other neurocognitive-related disease processes. ICU patients frequently develop post-ICU syndrome with a spectrum of chronic cognitive impairments reminiscent of AD; the persistent elevations of Aβx-40 plasma level in both ICU cohorts observed at time of admission to 7 days later may be a contributing factor. Considering that Aβx-40 has been shown to be the primary form of soluble Aβ excreted in urine [52], it will be important for future studies to determine whether increasing Aβx-40 plasma levels are due to a shift in excretion caused by kidney dysfunction. Of additional interest, sepsis causes long-lasting, trained innate immunity that increases Aβ-induced cognitive impairments in mice [53]. The mechanisms underlying Aβ switching from a protective antimicrobial function to a deleterious hyper-inflammatory function remains an outstanding question. Our observations raise the tantalising prospect that caspase-1 plays an integral role in regulating this Aβ functional switch, which will be examined in future studies.

A strength of our study design was the inclusion of an ICU cohort without evidence of infection, which controlled for any potential non-infectious factors associated with being in the ICU. There are, however, several limitations inherent to our study. Firstly, our patient cohort was of moderate size and from a single site pulmonary and critical care ICU. Therefore, in order to establish a plasma threshold for either caspase-1 and/or Aβx-40 with predictive clinical value, additional studies involving multiple centres is needed to increase cohort size and demographic representation. In addition, our healthy controls were skewed towards a younger Caucasian female demographic; however, days in the hospital or ICU were the only variables that were significantly correlated with age, and there were no significant correlations with sex or race. Secondly, we only obtained a 7dp admission sample from a subset of patients and no further outcomes post-discharge were assessed. Therefore, further studies will be required to determine whether these analyte levels are stable over time as patients are treated in the ICU, and, perhaps most interestingly, if these patients suffer from post-intensive care syndrome and other neurocognitive dysfunctions. Finally, our database did not include pathogen identities to facilitate additional causative agent correlations. These limitations notwithstanding, our findings have established a novel relationship between caspase-1 and Aβx-40 that may be important in regulating the short-term host inflammatory response to infection as well as the long-term outcomes in patients hospitalised with sepsis.

## Supplementary material

10.1183/23120541.00572-2023.Supp1 **Please note:** supplementary material is not edited by the Editorial Office, and is uploaded as it has been supplied by the author.
Supplementary material 00572-2023.SUPPLEMENT
