# Supplementary material for: Amyloid-β and caspase-1 are indicators of sepsis and organ injury
Source: ERJ Open Res. 2024 Feb 26;10(1):00572-2023. doi: 10.1183/23120541.00572-2023 (PMC10895426; doi:10.1183/23120541.00572-2023)
Supplement: Supplementary file 1 [file 00572-2023.SUPPLEMENT.pdf]

Supplemental Table 1. Distribution of Analytes Compared to Healthy Controls*

		Healthy Controls	ICU Non-Sepsis	ICU Sepsis
Caspase-1	% Below Median	50.00%	16.67%	5.26%
	% Above Median	50.00%	83.33%	94.74%
	p-value		<0.0001	<0.0001
IL-18	% Below Median	48.65%	44.44%	18.18%
	% Above Median	51.35%	55.56%	81.82%
	p-value		NS	<0.0001
$A\beta_{x\text{-}40}$	% Below Median	50.00%	50.00%	20.00%
	% Above Median	50.00%	50.00%	80.00%
	p-value		NS	<0.0001

*ICU patient data distribution was compared to that of the healthy controls and represented at percent of data points above or below the healthy control median using a two-sided 1 proportion z-test with $p < 0.05$ considered significant. NS = not significant.

Supplemental Table 2. Analyte Variance*

Analyte	Healthy Control	ICU Non-Sepsis	ICU Sepsis	p-value
Caspase-1	n^= 54<br>23.719#	n= 36<br>89.872	n= 76<br>741.309	<0.0001
IL-18	n= 37<br>84.638	n= 36<br>408.982	n= 77<br>2291.827	0.0031
Aβ_{x-40}	n= 54<br>131.606	n= 36<br>134.405	n= 75<br>213.089	<0.0001
Aβ_{x-42}	n= 15<br>150.976	n= 30<br>16.106	n= 55<br>93.483	0.0002
A$\beta_{x-42/x-40}$	n= 15<br>0.220	n= 30<br>0.145	n= 54<br>0.127	0.0056
TNF-α	n= 35<br>131.544	n= 22<br>25.833	n= 54<br>268.141	0.0011
IL-6	n= 33<br>48.543	n= 35<br>109.424	n= 75<br>2964.922	0.0457
IL-8	n= 36<br>60.780	n= 33<br>106.909	n= 65<br>1011.525	0.0419
IFN-α	n= 24<br>24.887	n= 21<br>12.083	n= 52<br>127.086	0.0030

*A majority of these analytes tested displayed a high level of variance. Levene's test with a $p < 0.05$ was used to identify analyte variants that are statistically different.
^Number of observations
#Standard deviations

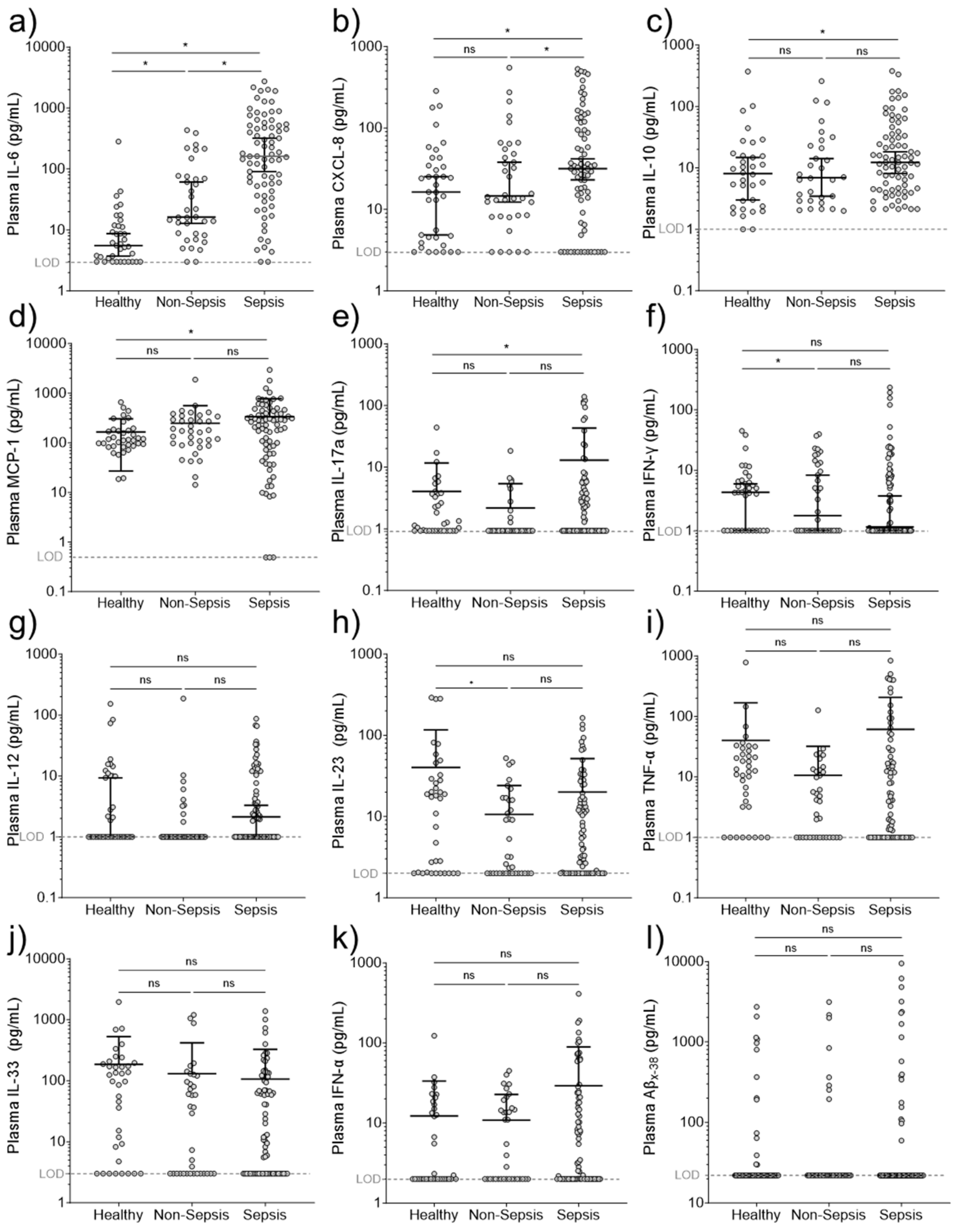

Supplemental Figure 1. Other plasma cytokine analytes. * $p<0.05$ was considered significant when comparing medians using a Steel test.

Characteristic	Pulmonary Sepsis (n = 39)	Non-Pulmonary Sepsis (n = 38)
**Average Age - yr. (range)**	60.1 (21-91)	57.6 (20-87)
**Male sex - no./total. (%)**	20/39 (51.3%)	22/38 (57.9%)
**Female Sex -no./total. (%)**	19/39 (48.7%)	16/38 (42.1%)
**Race - no./total. (%)**		
**White**	18/39 (46.2%)	17/38 (44.7%)
**Black**	21/39 (53.8%)	21/38 (55.3%)
**Intubated - no./total. (%)**	25/39 (64.1%)	7/36^ (19.4%)
**Vasopressor - no./total. (%)**	9/39 (23.1%)	12/38 (31.6%)

^Intubation status of 2 Sepsis Non-Pulmonary patients are unknown.

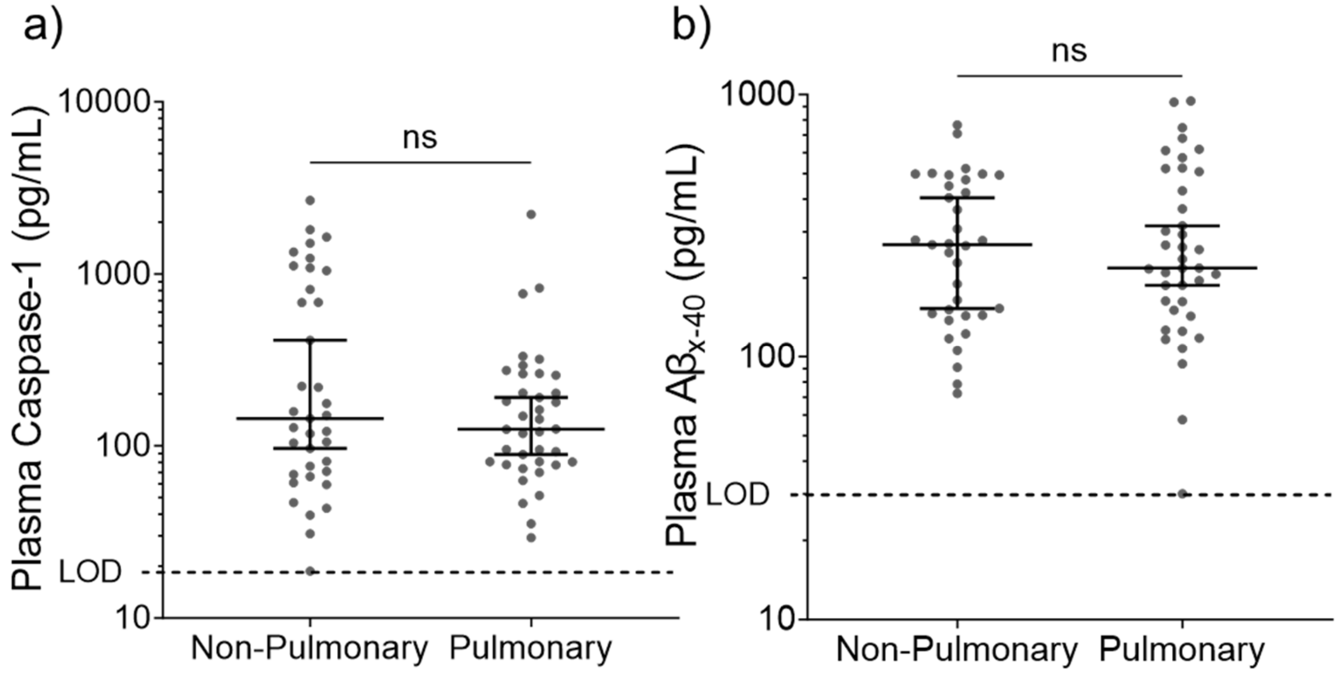

Supplemental Figure 2. The origin of the sepsis infection does not significantly alter cytokine levels. When the sepsis group is subdivided into either sepsis of a pulmonary origin or sepsis of a non-pulmonary origin, **a)** Capsase-1 and **b)** Aβ_{x-40} are not significantly different between the two sepsis groups.

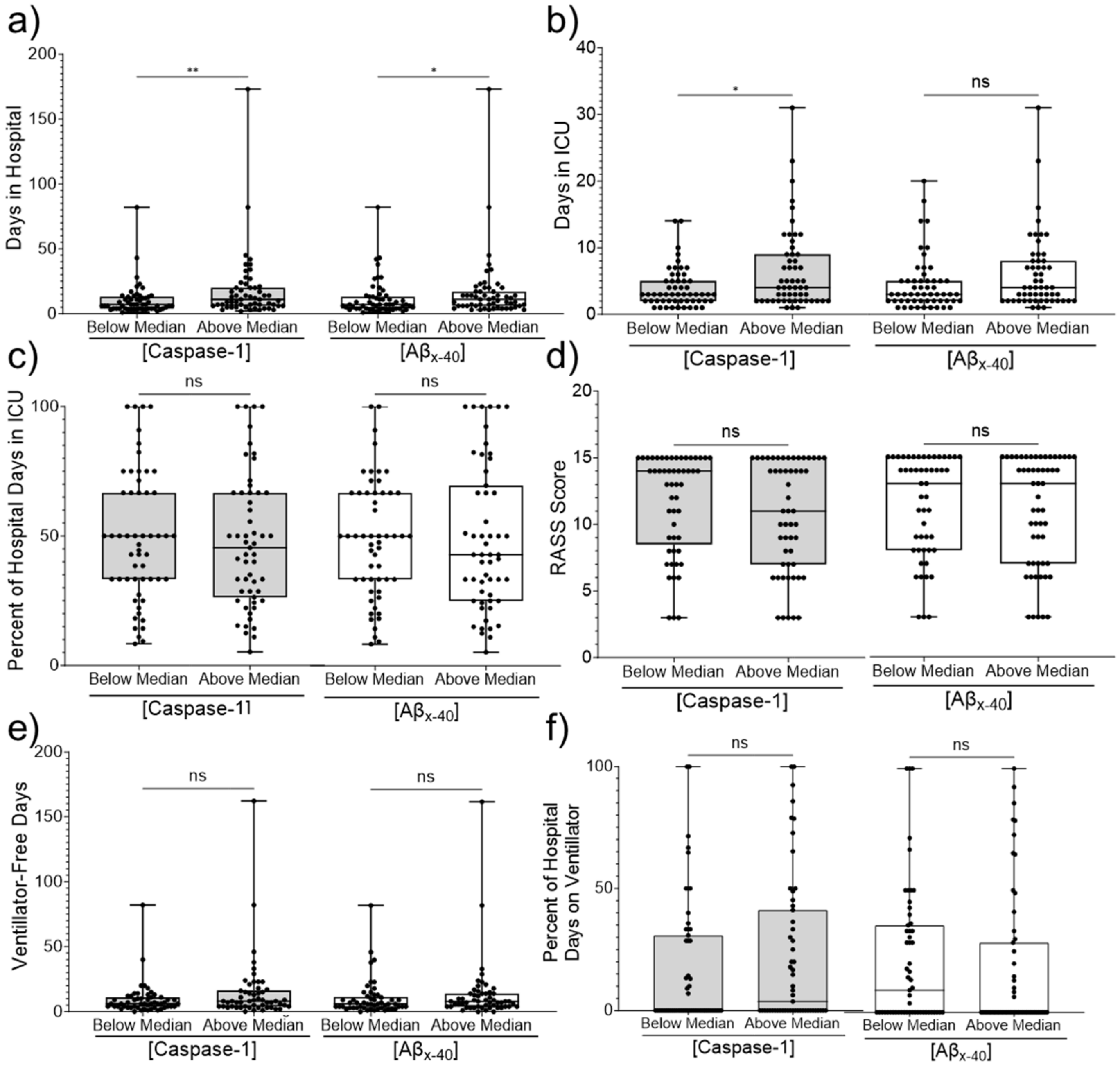

Supplemental Figure 3. Other patient outcome data. * $p<0.05$ or ** $p<0.01$ were considered significant when comparing medians using the Mann-Whitney test.
